# Entropy-Based Semi-Fragile Watermarking of Remote Sensing Images in the Wavelet Domain

**DOI:** 10.3390/e21090847

**Published:** 2019-08-30

**Authors:** Jordi Serra-Ruiz, Amna Qureshi, David Megías

**Affiliations:** Internet Interdisciplinary Institute (IN3), Universitat Oberta de Catalunya (UOC), CYBERCAT-Center for Cybersecurity Research of Catalonia, 08860 Castelldefels, Barcelona, Spain

**Keywords:** entropy, tampering detection, image forensics, image authentication, semi-fragile watermarking, wavelet Transform, hyperspectral images

## Abstract

This article presents a semi-fragile image tampering detection method for multi-band images. In the proposed scheme, a mark is embedded into remote sensing images, which have multiple frequential values for each pixel, applying tree-structured vector quantization. The mark is not embedded into each frequency band separately, but all the spectral values (known as signature) are used. The mark is embedded in the signature as a means to detect if the original image has been forged. The image is partitioned into three-dimensional blocks with varying sizes. The size of these blocks and the embedded mark is determined by the entropy of each region. The image blocks contain areas that have similar pixel values and represent smooth regions in multispectral or hyperspectral images. Each block is first transformed using the discrete wavelet transform. Then, a tree-structured vector quantizer (TSVQ) is constructed from the low-frequency region of each block. An iterative algorithm is applied to the generated trees until the resulting tree fulfils a requisite criterion. More precisely, the TSVQ tree that matches a particular value of entropy and provides a near-optimal value according to Shannon’s rate-distortion function is selected. The proposed method is shown to be able to preserve the embedded mark under lossy compression (above a given threshold) but, at the same time, it detects possibly forged blocks and their positions in the whole image. Experimental results show how the scheme can be applied to detect forgery attacks, and JPEG2000 compression of the images can be applied without removing the authentication mark. The scheme is also compared to other works in the literature.

## 1. Introduction

The interest in remote sensing images has increased in the last few years. New applications of this type of images are continuously being reported. For example, they are used for finding water in remote planets (NASA missions), for water pollution control in the oceans (e.g., detection of plastics, seaweeds, oil, etc.), or for high precision farming, among others [1,2]. Both the equipment and the system for obtaining this kind of images are expensive, since either an aircraft (for small areas) or a satellite (for larger regions) are required. Therefore, the economic cost and the true signatures of the materials detected in the surface should be protected upon purchase of these images by a third party.

Internet and digitalization have favored the distribution of all file types, which has given rise to the problem of handling authorized and illegal copying (piracy) and redistribution of multimedia contents. In general, to prevent piracy and address the issues faced by the proliferation of digital media (i.e., video, audio or image files), watermarking techniques have been developed during the last three decades. In many application scenarios, the watermarks are covertly embedded into the digital media to provide content integrity and data ownership. Most image protection methods can be classified into two following categories based on the requirements: fragile (or semi-fragile) watermarking, and robust watermarking. For content authentication, it is usually required not only to confirm the source of the digital media, but also to verify that it has not been tampered in any case. In case of forgery, the localization of the tampered areas is also typically demanded. This kind of application is usually obtained through fragile or semi-fragile watermarking.

Modification of digital contents and redistribution without permission are now easier than ever and, therefore, research in content protection [3] and distribution has increased. Many applications require a system both to prove the source of digital content and its integrity. Different fragile and semi-fragile watermarking methods have been proposed to detect image forgeries. Some of these methods can identify the area of the images that has been modified. Fragile schemes [4,5] can detect all image modifications, even if they are very small and affect a reduced number of pixels (or a single pixel). On the other hand, semi-fragile schemes permit a moderate modification of the original image, such as some degree of lossy compression or the application of a simple filter. Fragile watermarking does not allow any kind of image processing operation. Semi-fragile watermarking offers more flexibility such as distribution of a lossy-compressed version of the image at a reduced price, while preserving the property of forgery detection and localization.

Semi-fragile methods prevent applying the embedding process to all the versions of the same image, thus reducing the production and distribution costs for different versions of the same image with different quality factors. A potential buyer may obtain a lower-quality version that allows him/her to have the certainty that the image has not been modified and prove that it is part of the image that he/she needs. Then, the buyer can purchase the best quality image being sure that it is an original non-tampered image.

In contrast to fragile or semi-fragile watermarking, robust watermarking schemes allow detection of the embedded watermark even after strong image processing operations. Robust watermarking purposes to resist attacks that attempt to remove or destroy the watermark through various kinds of manipulations, e.g., compression, noise addition, filtering, geometrical attacks (rotation, scaling), etc. However, robustness and imperceptibility are conflicting requirements for any watermarking application, i.e., to enhance robustness, the strength of the watermark needs to be increased at the expense of loss in imperceptibility, and vice versa. Therefore, the optimal trade-off between robustness and transparency properties must be achieved while designing a watermarking scheme. Robust watermarking methods have proven successful to protect some types of images in several ways, such as detection of tampering or manipulations made in the images with an aim to produce a forged copy [6,7].

For remote sensing images, semi-fragile watermarking schemes are often preferred. In semi-fragile schemes, the watermark is preserved against minor modifications (e.g., near-lossless compression), whereas stronger manipulations (e.g., copy-and-replace attacks, cropping, or lossy compression, etc.) damage the embedded watermark, and can be easily detected. Most of the existing semi-fragile watermarking schemes are designed for grayscale or RGB images, and these can be applied to each separate spectral band of remote sensing (hyperspectral or multispectral) images.

Tampering detection can also be obtained using hashing techniques. In the watermarking schemes, the image to be protected is modified during the embedding process, whereas, the hashing schemes consist of obtaining a value related to the image that can be checked at the receiver side. Hashing schemes, hence, do not introduce distortion in the protected image, and this is a clear advantage compared to watermarking. Although it is possible to develop robust or semi-robust hashing schemes that yield the same numerical value in case of minor modifications, they do not typically allow for tamper localization. If tampering localization is required, a hashing algorithm can be applied blockwise, but the amount of side information will significantly increase, since a different hash value would be required for each block. In addition, this side information needs to be protected, e.g., by using digital signatures, to prevent malicious attacks. In contrast, watermarking usually requires only a short secret key both at the embedder and the receiver sides to authenticate the image. The authentication mark is included in the image and does not need to be distributed separately. Furthermore, a semi-fragile watermarking scheme can be applied to various images with the same secret key. Hence, no separate side information needs to be distributed (other than the secret key) together with the image, which constitutes a remarkable advantage of watermarking compared to hashing techniques. If no modification of the host image is required, reversible watermarking [8] or zero-watermarking [9] schemes can be used instead of traditional watermarking.

Different works in the literature propose watermarking methods for satellite images. A semi-fragile watermarking scheme is proposed in [10] in which a hyperspectral image is used for embedding the watermark to provide tamper detection by using vector quantization approach. The hyperspectral image is segmented into three-dimensional blocks of pre-determined size, which determines the spatial resolution of both embedding and detection algorithms. A tree with an endmember of the image is built for each block. The least significant bits (LSBs) of these endmembers are removed to provide protection from possible near-lossless compression attacks. An iterative algorithm is applied to each block until the resulting block meets the specific criterion. The image is changed according to a secret key that yields a different criterion for each block to avoid copy-and-replace attacks. However, the proposed scheme needs to save the secret key for each sub-block as an auxiliary information.

A robust multispectral image watermarking technique based on the discrete wavelet transform (DWT) and Tucker Decomposition (TD) is proposed in [11]. In the proposed technique, DWT is applied to each spectral band of a multispectral image to obtain sub-bands, and then, TD is applied to the approximate sub-bands to construct a third order tensor. The watermark is embedded into the elements of the last frontal slices of the core tensor with the smallest absolute value. However, the last frontal slice of high-order tensor is not stable enough for persevering correlations of RGB channels of the image.

In [12], the authors present a copyright protection scheme based on the DWT for raster images. A 2-D DWT is applied on each channel (R, G, and B) of a raster image, and two sub-bands (level-3 low and high-frequency coefficients, i.e., LL3 and HH3) are used for embedding the watermark. The scheme is evaluated against JPEG compression for different quality factors, and the results indicate that only for higher quality factors, the proposed method can correctly extract the watermark. However, the scheme is not evaluated against tampering attacks such as copy-and-replace or image splicing attacks.

Reference [13] presents a histogram shifting-based data hiding method for embedding the visible light image in the Fourier transform infrared (FTIR) spectral image. Before embedding, the payload data is encoded using error-correcting codes. Two different approaches for error correction are proposed to enable corrections of the payload data after extraction. Tamper proofing is provided by applying Hash-Based Message Authentication Codes (HMAC) to generate an authentication tag to be embedded into the FTIR spectral image. Given the proper quantization settings, the proposed technique guarantees high capacity and good fidelity between the original FTIR structure and its marked version. However, the proposed method is time-consuming, the quality of the extracted visible light image is not good, and bit errors cause problems in deletion channel correction encoding.

A robust blind watermarking scheme has been proposed in [14] for multispectral Landsat images. A subset of bands is selected from the available ones using a secret key. Then, quadtree decomposition-based homogeneity analysis of the selected bands is carried out using a user-defined threshold value, which is stored as a second secret key. The watermark is encoded using convolution encoding, and the encoding rate is kept as a third secret key. The blocks for embedding the encoded information are selected by using a randomly generated fourth secret key. The extraction process is blind and requires four secret keys, known only to the authentic owner. Though the scheme provides security against common signal processing attacks (e.g., filtering, noise addition, rotation, etc.), it does not address tampering attacks such as copy-and-replace or image splicing attack.

In [15], a semi-fragile watermarking scheme is proposed for tamper localization and recovery of satellite images. The scheme uses secret keys to generate a pseudo-random binary matrix as the authentication watermark for locating tampered locations, while recovery watermark is designed for restoring the tampered content. The Contourlet Transform is applied to the remote sensing image, and the authentication watermark is embedded in the coefficients that have the maximum value of all sub-bands. The restoration of tampered remote sensing image can be performed efficiently, given the request for data accuracy is not too rigid. The security against tampering attacks is mentioned, but it does not provide the names of the attacks carried out on the watermarked remote sensing images.

Reference [16] presents a novel one-pass framework based on a modification of prediction errors scheme, which enables data hiding and lossless compression operations simultaneously on hyperspectral images collected through remote sensing facilities. The scheme employs three-dimensional predictors to exploit the redundancies of hyperspectral images. The proposed scheme has limited computational complexity and storage capacity, and does not provide security against lossy compression.

In this paper, a novel semi-fragile watermarking scheme is particularly designed for remote sensing images. The proposed scheme does not embed the watermark in a single band, but selects a group of relevant spectral bands across the frequency spectrum and constructs a vector-based representation of the image to be protected. The relevant bands can be selected according to their particular relevance for specific applications or they can be selected by using a regular sampling. It is also possible to select all the spectral bands at the price of a larger computational burden.

Many fragile or semi-fragile watermarking schemes proposed for multi-band images have either considered a single band or processed each band separately  [17,18]. Embedding the information in each band separately is usually simpler, but can lead to uneven modifications of different spectral components, introducing significant distortion in the spectral signature of the pixels. On the other hand, the method proposed in this paper works with vectors formed with different spectral frequencies and, thus, avoids this kind of spurious distortions. The proposed method preserves the shapes of the signatures and, thus, prevents misclassification of the image, which is extremely relevant for this kind of application.

The proposed watermarking scheme divides the image into three-dimensional (where two dimensions represent spatial features and the third one represents spectral features) blocks of a variable size. The size of these blocks decides the spatial resolution of the tampering localization process: the larger the blocks, the less accurate the tampering localization. For each block, a tree of endmembers (the values obtained by the remote sensor for each pixel) is constructed. These endmembers are transformed using the DWT to provide robustness against near-lossless compression. Then, the tree corresponding to each block is modified using an iterative TSVQ process until a particular entropy value is obtained for the tree. This entropy value represents the particular watermark for each block. Copy-and-replace attacks will lead to a different entropy value and, thus, this kind of malicious modification will be detected by the watermark detection algorithm. The particular entropy value is chosen according to a secret key, which selects the final structure of the TSVQ tree and, thereby, introduces distortion into each block.

The remainder of this paper is organized as follows. Section 2 presents an overview of a few techniques used in current encoding systems. Section 3 describes in detail the mark embedding and the mark detection methods. The results of the proposed watermarking scheme for the selected experimental corpus are presented in Section 4. A comparative analysis between the suggested scheme and other similar state-of-the-art watermarking schemes in terms of embedding strategy, PSNR of the marked remote sensing images, and tamper localization is also presented. Finally, Section 5 summarizes the most relevant conclusions of this work.

## 2. Background

This section presents an overview of the three fundamental concepts used in the proposed scheme, namely remote sensing images, lossy compression, and vector quantization.

### 2.1. Remote Sensing Images

**Figure 1:**
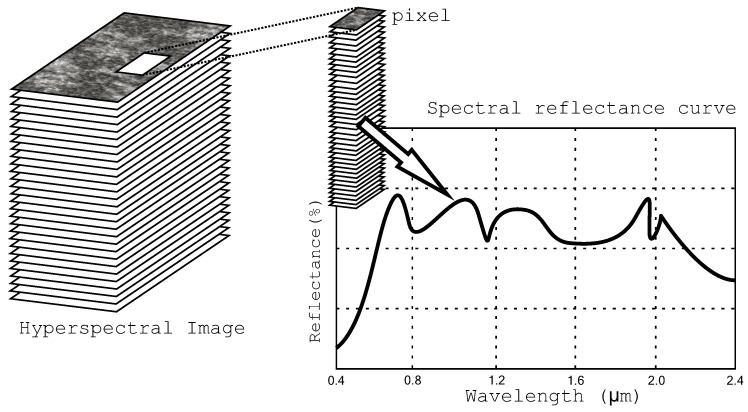
An example of a signature curve for a pixel.

A remote sensing image stores information about a broad area of Earth’s surface. Each pixel of this image is represented by a set of values, named signature or endmember, obtained by a special sensor for different frequencies of the light spectrum (frequency bands). The signature of each pixel of the remote sensing image is associated with the light reflectance of the different materials found within the coverage area e.g., water, dry soil, green vegetation, or minerals, etc. Figure 1 shows an example of a signature curve for a pixel of the remote sensing image.

**Figure 2:**
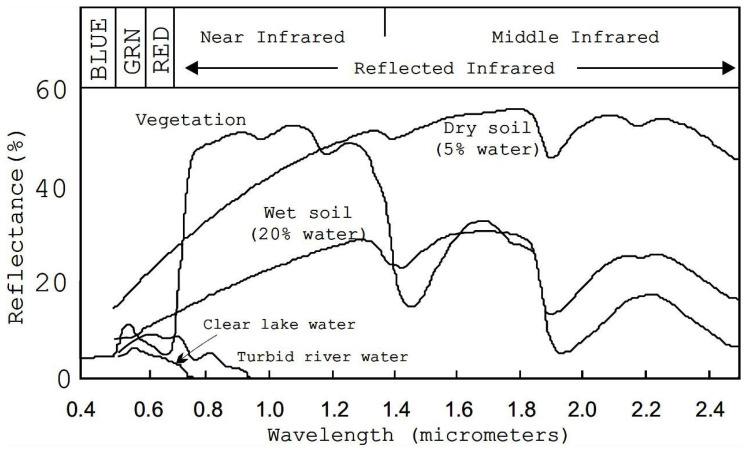
Representative reflectance spectra of different materials.

The graphical illustration of different signatures presented in [19] is shown in Figure 2. In this figure, different signatures curves of light reflectance for several common Earth surface materials, namely vegetation, dry and wet soil, clear lake water, and turbid river water are presented.

Managing these large-sized images presents a significant challenge for researchers. A typical hyperspectral image that covers a geographical area of a few kilometers may contain millions of pixels. Each of these pixels consists of hundreds of contiguous and narrow spectral bands covering typically the visible to short infrared wavelength range (determined by the type of deployed sensor). For example, Airborne Visible/Infrared Imaging Spectrometer (AVIRIS) [20] images consist of 224 contiguous spectral channels (bands), and are typically represented with 16-bit (0–65,535 range) precision. Multispectral images are relatively low spatial resolution images and generally consist of 4 to 10 spectral bands, e.g., a Landsat image [21] is a multispectral image that uses 8 bands/pixel with a precision of 8 bits (0–255 range). The number of bands in a remote sensing image varies depending on the sensor system. The number of bands determines the names of the remote sensing image, i.e., multispectral, hyperspectral, or ultraspectral.

A lossy compression technique [22,23] is used to reduce the amount of data needed to represent the image. The term “lossy” implies that some data from the original image is permanently lost. It is an irreversible process, i.e., upon compressing the image, and subsequently decompressing it, the recovered image closely resembles, but is not an exact duplicate of the original image due to the removal of some information. While some particular applications do not permit any kind of information loss, there are many other applications in which lack of exact reconstruction is not a problem. However, it is necessary to be able to control distortion introduced due to the lossy compression process, i.e., the distortion must be less than a predefined threshold to avoid damaging relevant information.

### 2.2. Lossy Compression of Remote Sensing Images

In lossy compression methods, non-significant information that is of little importance in reconstruction of the image is removed. This is a critical issue in lossy image compression, since the elimination of data should be decided by the user depending on his/her purpose of use. Different image quality criteria [24] can be defined based on the desirable goals. Preliminary experiments [25,26] show that it is possible to achieve relatively high compression ratios without removing critical information.

The general coding scheme must be altered according to the specific characteristics of the source to maximize both compression ratio and image fidelity. In this case, we must deal with a 3D remote sensing image, which has two spatial dimensions and one spectral dimension. An ideal compression method exploits redundancies in both the spatial and spectral dimensions. The approach of encoding each band independently without considering the spectral redundancy between bands is clearly suboptimal (low encoding performance), while applying 3D-coding schemes that do not consider the difference between spatial and spectral dimensions. In the literature, many 3D transformation schemes have been proposed to decorrelate spatial and spectral redundancy, e.g., a survey [27] presented on hyperspectral image compression uses vector quantization for lossy compression purpose, and processes all bands simultaneously.

### 2.3. Discrete Wavelet Transform

The Discrete Wavelet Transform (DWT) is a mathematical operation that converts an image into four sub-bands of information—HH, HL, LH and LL—that contain approximation (low-frequency) coefficients (LL), detail middle-frequency coefficients (HL and LH), and detail high-frequency coefficients (HH). This transform makes it possible to separate the coefficients that contain most of the image information (basically LL) from those that contain higher frequency details (HL, LH, and HH).

This transform is exploited by some compression algorithms, such as JPEG2000 [28]. To design a watermarking scheme that is robust against JPEG2000 compression, it is a good idea to embed the watermark in the approximation coefficients (LL), whereas the other three sub-bands are left unaltered. Similarly, if a watermarking system is to be robust against standard JPEG compression [29], we should consider the DCT transform in 8×8 blocks of pixels, exactly as the JPEG compressor works.

As already remarked, remote sensing images have a relevant economic cost, and it is advisable to use a compression algorithm that introduces less distortion. Hence, JPEG2000 compression is much more suitable than JPEG compression for these kinds of images. This is the reason for choosing the DWT as the mathematical transform for this application.

### 2.4. Watermarking

Watermarking [30] is a process of imperceptibly embedding a particular information (watermark) into a cover object (e.g., a remote sensing image) to generate a watermarked copy of the same object. The watermark embedding process involves the modification of the original media content to embed the watermark. In many watermarking schemes, a common (secret) key is used in both the watermark insertion and extraction processes. At the detector/extractor end, the same secret key used in embedding is required for the detection of the watermark in the test object. In case of a blind detection technique, the original unmarked content is not needed.

A semi-fragile scheme is well-suited for content authentication due to its ability to tolerate minor modifications (e.g., near-lossless compression) and reveal significant manipulations (e.g., copy-and-replace attack, elimination of excessive information through cropping, or lossy compression, etc.). In contrast, robust watermarking schemes are well-suited for copyright protection because of the ability to resist various kinds of manipulations, e.g.,  compression, noise addition, filtering, geometrical attacks (rotation, scaling), etc. In general, enhancing watermark robustness requires more image distortion in the marked image, which results in lower imperceptibility. Thus, it is important to achieve a convenient trade-off between robustness and transparency properties while designing a watermarking scheme. Many robust watermarking schemes [6,7] have been proved effective in proving content ownership and preserving the integrity of images.

Most of the proposed schemes in remote sensing imaging applications use semi-fragile watermarking systems to detect unauthorized minor (e.g., near-lossless compression) or major modifications (e.g., copy-and-replace attack) in the image. Several existing semi-fragile watermarking applications are designed for monochromatic images, and therefore, can be easily applied to remote sensing (multispectral or hyperspectral) images by processing each band separately.

### 2.5. Vector Quantization and Tree-Structured Vector Quantization

Vector Quantization (VQ) [31] is based on the Shannon’s rate-distortion theory, which states that optimal image compression can be achieved by coding vectors instead of scalars. Shannon distortion-rate function [32] characterizes the minimum achievable rate at a given bit rate by any vector quantizers (multidimensional) under the given distortion measure and a given source (with all its statistical properties known). Shannon’s rate-distortion theory addresses the problem of determining the minimum amount of entropy (or information) required to be communicated over a channel, so as to enable the receiver to achieve approximate reconstruction of the source signal without surpassing a specified distortion measure. Therefore, applying VQ compression results in minimum image distortion (locally) for a given compression ratio. However, the computational and storage requirements of VQ compression become prohibitive as the dimensionality of the vectors increases. Therefore, there is a need to explore suboptimal compression methods for practical applications.

A suboptimal strategy, TSVQ, starts with the initial centroid as the codebook, i.e., a tree with a single leaf, and then Mean Square Error (MSE) is applied as the quality evaluation criterion. Depending on the criterion, if there is chance of improvement, the leaf node with the highest value of the distortion is divided into two similar centroids. Then, new centroids are computed by applying the Linde–Buzo–Gray algorithm [33]. This process goes on until a stopping quality criterion is satisfied or a perfect tree is constructed. Given a large image and a huge amount of training vectors results in a highly unbalanced tree with a large depth. However, with large enough subtrees, it is possible to find subtrees suitable for embedding a watermark.

Finally, with the selected subtree, the original image is coded by replacing each original vector with the closest centroid. This centroid contains all the elements in the leaf where the original vector lies. This selection consists of traversing the tree from the root node and choosing the closest centroid until a leaf is reached. Coding a *selected* tree leads to more efficient encoding time than the generation of the initial tree *T*.

## 3. Semi-Fragile Watermarking Scheme

In this section, the proposed watermarking scheme is described. Section 3.1 discusses the embedding method based on tiling, DWT, and TSVQ, while in Section 3.2, the tampering detection and localization process is detailed.

### 3.1. Mark Embedding Process

To explain the proposed scheme, we have considered here a three-dimensional hyperspectral image *I* of size M×N×b samples, where *b* denotes the number of bands. The image *I* is segmented into different blocks or tiles of size $W_{i}\times H_{i}\times b^{\prime }$, where $W_{i}\leq M$ and $H_{i}\leq N$ denote the pixel sizes of each block (different for each block) and $b^{\prime }\leq b$ stands for the number of selected bands. This block division allows detection of specific tampered regions in the attacked images. Using blocks of different sizes makes the scheme secure against some attacks based on analyzing the properties of the blocks by an attacker who may know the watermarking process. If the attacker attempts to recreate the properties of a forged area, he/she must know the exact tiling of the image. Keeping the tiling secret is thus, an additional measure of security.

**Figure 3:**
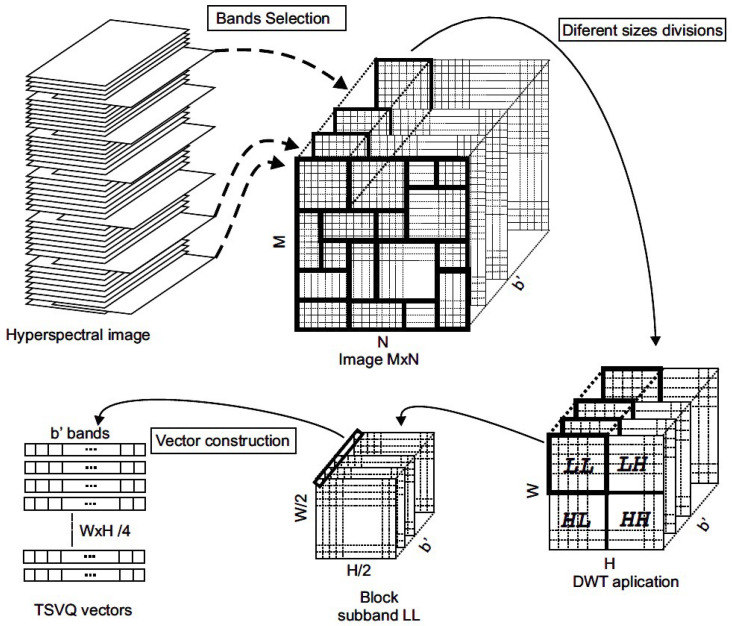
Generation of the signature vectors for TSVQ.

As detailed in Figure 3, the original image of size 512×512 is segmented into smaller blocks of various sizes, with each block being marked separately. The specific tiling information for each image, i.e., the size and position of each block must be recorded and transmitted to the detector as part of the secret key. Although choosing a manual tiling of the image is possible, in this article, we propose an automatic tiling process to obtain more homogeneous regions. The automatic tiling process is described below.

This process is run for all the selected bands $b_{k}$. The selected band is divided into mini blocks of size 32×32 samples. These blocks are compared with their neighbors in order to build larger blocks of the following possible sizes $W_{i},H_{i}\in \left\{32,64,96,128\right\}$, hence the smallest possible block size is 32×32 and the largest possible block size is 128×128. The unassigned blocks of size 32×32 samples are grouped in larger contiguous super-blocks of 128×128 samples (or 4×4 sub-blocks). For each super-block, the average of the samples of its 16 sub-blocks $B_{i,j}$ with i,j∈{1,2,3,4} is obtained and compared with that of a possible block $B^{\prime }$ of size 32j×32i containing $B_{i,j}$. This process is summarized in Algorithm 1.

**Figure 4:**
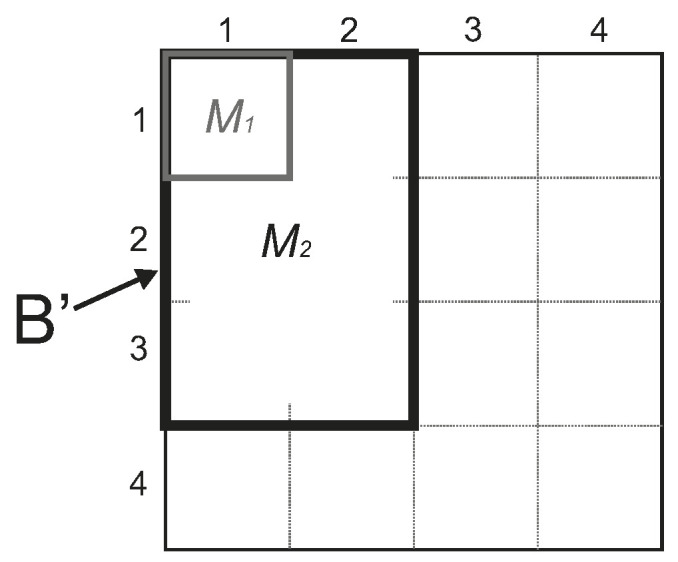
Candidate block $B^{\prime }$ including sub-block $B_{3,2}$.

Figure 4 illustrates how the average of the samples of the sub-block $B_{3,2}$ is compared with that of a possible block $B^{\prime }$ of size 64×96 samples. If the difference between these averages is lower than a given threshold θ, then the candidate $B^{\prime }$ is selected as a possible block. After the process is completed, the largest possible block $B^{\prime }$ fulfilling this condition is selected. More precisely, the averages $M_{1}$ and $M_{2}$ are obtained as follows:

$$\begin{array}{c} M_{1}=\frac{\sum_{n=1}^{32}\sum_{m=1}^{32}B_{i,j}\left(n,m\right)}{32\times 32},\\ M_{2}=\frac{\sum_{n=1}^{32i}\sum_{m=1}^{32j}B^{\prime }\left(n,m\right)}{32i\times 32j}, \end{array}$$

where *i* and *j* are the coordinates of the sub-block $B_{i,j}$ being analyzed, $B_{i,j}\left(\cdot ,\cdot \right)$ are the samples of the mini-block and $B^{\prime }\left(\cdot ,\cdot \right)$ are the samples of the candidate block. The block $B^{\prime }$ is selected as eligible if it turns out that $|M_{2}-M_{1}|\leq \theta$. Finally, the largest eligible block $B^{\prime }$ is selected and included in the tiling for the chosen band.

In this process, $b^{\prime }$ alternative tilings, one for each the $b^{\prime }$ selected bands, are produced. Among these $b^{\prime }$ alternative block divisions, we select the one that provides the largest number of blocks. This means that the average block size for the selected tiling is the smallest among the $b^{\prime }$ alternative ones, since the average block size is simply the size of the image divided by the number of blocks. The block division (i.e., the coordinates of the upper left corner and the width and height of each block) are kept as part of the secret key of the method and is required in the watermark detection process.

**Figure 5:**
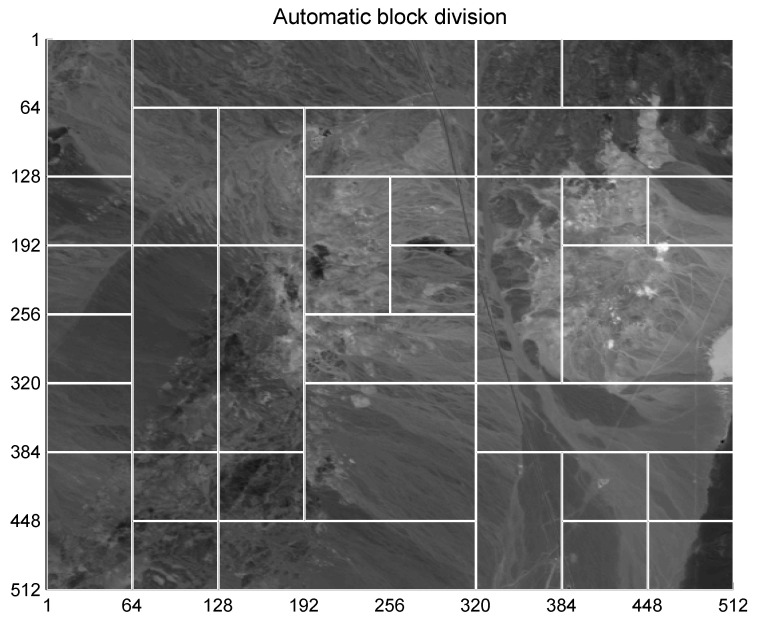
Block division obtained after applying the tiling process.

Figure 5 shows an example of automatic block division using this method. As it can be seen, generally, there are larger blocks for homogeneous regions (low entropy regions) and smaller blocks for more variable areas (high entropy regions). Larger blocks are more convenient to reduce computations and achieve a more even distortion, whereas smaller blocks provide specific protection for regions of more interest.

**Algorithm 1** Tiling process
For all the bands of the whole image do:(a)Split the band into 32×32 samples blocks.(b)Calculate the average value $M_{1}$ of each sub-block $B_{i,j}$ (Figure 4).(c)For all the $B_{i,j}$  do:i.Calculate the 16 average values $M_{2,k}$ of  4×4 sub-blocks (where k=1,…,16).ii.Compare the averages $M_{1}$ and $M_{2,k}$ (for k=1,…,16).iii.Select the largest sub-block $M_{2,k}$ such that the average difference is lower than θ (threshold).Select the final distribution of blocks from the band that leads to a larger number of blocks.


Once the tiling process is completed, the DWT is applied to each block to achieve robustness against near-lossless compression. The LL sub-band of the DWT is then used to construct TSVQ vectors. These vectors are formed by substituting the coefficients of the LL sub-band of each block with very similar values obtained from the leaves of a TSVQ tree with minimal distortion. This construction of vectors for each block enforces a particular property, i.e., entropy of the tree, which will be verified at the detector end. Then, the Breiman, Friedman, Olshen, and Stone (BFOS) algorithm [34] is applied to the generated tree to prune it with the selected criterion, which results in the generation of entire subtrees in the compression ratio-distortion curve. The entropy of the resulting TSVQ tree determines the subtree that is a pre-requisite for obtaining the specific compression of the image block. It is worth pointing out that the compression ratio does not consider the individual bands. Instead, the signatures as a whole (in fact, the selected bands $b^{\prime }$ within the signature) are considered, since a vector quantization approach is used in the proposed scheme. Therefore, the proposed scheme is different from other state-of-the-art semi-fragile watermarking methods that process each band separately. Figure 3 summarizes the embedding process.

A Pseudo-Random Number Generator (PRNG) is used to select different entropy (the marking property) for each of the tiles. The seed of PRNG is also needed in the detection process. Therefore, this seed constitutes a part of the secret key (combined with the specific tiling of the image). The criterion (entropy value) is calculated in the following manner:

(1) Entropy of the block =R−v·h,

where *R* is a reference value, *v* is a *n*-bit value between 0 and $2^{n-1}$ selected by using the PRNG, and $h=2^{-n}$ is a quantization step. The value of *n* is selected in such a way that $2^{n}$ is greater than or equal to the number of blocks of the image. The PRNG sequence is chosen to provide different values for each block. If a value is repeated from a previous block, it is discarded and another one is generated.

Then, using the selected TSVQ criterion, the LL sub-band of each block of the original image is compressed with a different compression ratio. This new $LL^{*}$ sub-band is processed with the TSVQ process again by using the same parameters as in the first iteration. This process is iterated until the resulting block satisfies the selected property and produces a modified $LL^{\prime }$ sub-band that is used in the marked image.

Regarding a manipulated marked image, a substantial amount of change made in any single band or a small number of bands is considered unacceptable in remote sensing images due to the fact that these unauthorized changes result in introducing an uneven change in the spectral signature. Only those manipulations that affect the whole signature, such as near-lossless compression, are accepted up to a certain threshold.

The potential tampering attacks, such as copy-and-replace or image splicing (in which parts of another image are copied and pasted into the marked image), can be detected by choosing a PRNG sequence that determines the selection criterion. This criterion is used to choose the compression subtree in the pruning algorithm. Therefore, finding such a pattern that may reveal the properties of a watermarking scheme is very difficult, and thus, enables reduction of the chances of image manipulation or modification through use of another region of the same image.

It is worth noting that copy-and-replace attacks are very difficult to perform with the non-uniform tiling of the image. This is because an attacker is unlikely to guess the exact size and location of a particular block so as to replace it with a different one (and with the same entropy).

**Figure 6:**
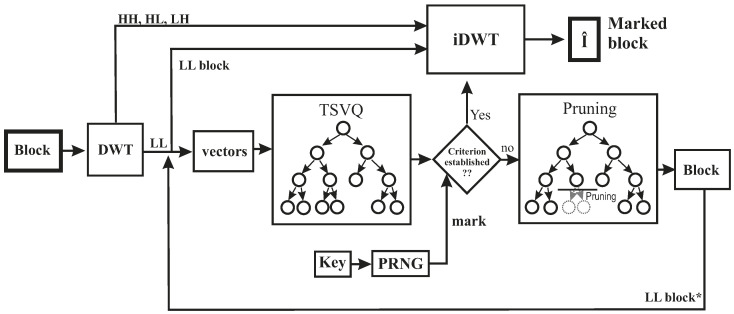
An overview of the embedding method.

In the suggested scheme, shown in Figure 6, it can be seen that entropy is used as a selection parameter for choosing a particular tree to build the marked $LL^{\prime }$ sub-band. The entropy of the compression tree of each block is computed by the pseudo-random sequence. Finally, a table with all the possible subtrees is generated by the BFOS algorithm. These subtrees lie in the convex hull, thus, providing better performance in terms of minimized distortion rate for a specified compression ratio. Typically, compression ratio and MSE are used to generate the convex hull; however, the BFOS algorithm can be applied by using any other criteria that could be convenient for applying watermarking or joint [35] compression and watermarking. After selection of a specific subtree for generation of the modified LL sub-band, the resulting marked content is obtained with its centroids yielding a specific value for the entropy that represents the embedded mark.

The result of the TSVQ process, i.e., the $LL^{\prime }$ DWT sub-band obtained with the centroids, is combined with the original LH, HL, and HH DWT sub-bands (kept in the first step) of the block. Then, an inverse DWT is applied to the combined sub-bands to generate the marked block. Lastly, the marked image is constructed by combining all the marked blocks.

As already mentioned, the secret key is generated by concatenating the tiling information, the seed of the PRNG, the chosen quantization step of the entropy, and the initial (reference) entropy value. However, the secret key is relatively a small (usually less than 2 or 3 KiB) and should be transmitted using a secured channel. The embedding process is summarized in Algorithm 2.

**Algorithm 2** Embedding process
0.Pre-process to suppress the noise of the sensors. This (optional) step may be achieved by slightly compressing–decompressing the image with some codec, such as JPEG2000, e.g., using the KaKaDu software of [36].1.Select the $b^{\prime }$ bands to be embedded.2.Run the tiling process of Algorithm 1 and save the positions and sizes of the selected blocks and build the image blocks as shown in Figure 3.3.Choose the property to use for embedding (e.g., entropy), its initial (reference) value and the quantization step.4.Select the seed and initialize the PRNG. Retain the seed as a constituent of the secret key.5.For all the blocks of the image selected in Step 2 do:(a)Apply the DWT and save the LH, HL, and HH sub-bands.(b)Use a pseudo-random number generator to generate a new random number. Then, verify that this number is not used in a previous block (otherwise generate a new one until no already used number results).(c)Choose the value of the marking property (e.g., entropy) according to the pseudo-random number obtained in the previous step (5b).(d)Start the TSVQ pruning process and choose a subtree satisfying the property obtained in the previous step (5c).(e)Generate a new sub-band $LL^{*}$ using the subtree obtained in the previous step (5d).(f)Check if the property chosen in Step 5c is satisfied with the tree built using the $LL^{*}$ sub-band obtained in Step 5e. If this property is not fulfilled, go back to Step 5d and use the new $LL^{*}$ sub-band. If the property is achieved, let $LL^{\prime }:=LL^{*}$.(g)Apply the inverse integer DWT to the resulting $LL^{\prime }$ sub-band combined with the original DWT sub-bands, i.e., LH, HL, and HH, stored in Step 5a.6.Build the final marked image by combining all the marked blocks (according to the saved sizes and positions) and the bands that were not selected in Step 1.


### 3.2. Tampering Detection and Localization

**Figure 7:**
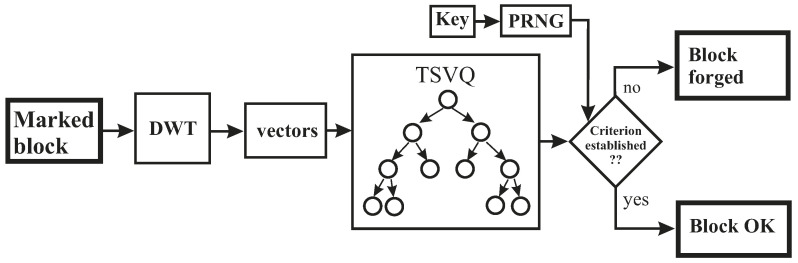
An overview of the detection method.

The watermark detection process is similar to the watermark embedding process. The mark can be detected without the help of the original image, thus implying a blind detection. The detection process is depicted in Figure 7 and Algorithm 3.

First, the same bands used during embedding process are extracted from the test image, and the tiling process is applied using the secret key. After that, the DWT is applied to each individual band of each block, and the TSVQ tree is built using the LL sub-band of each block. Next, we must verify if the obtained TSVQ tree satisfies the criterion provided by the pseudo-random sequence that is generated by using the seed contained in the secret key.

**Algorithm 3** Tampering detection and localization
Retrieve the $b^{\prime }$ marked bands of the test image (this information is contained in the secret key).Retrieve the image tiling: block sizes and positions (this information is also stored in the secret key).Retrieve the property used for embedding (e.g., entropy), its initial (reference) value and the quantization step (also from the secret key).Retrieve the seed of the PRNG from the secret key and initialize it.For all the blocks built in Step b do:(a)Apply the DWT to obtain the sub-bands LL, LH, HL, and HH for that block.(b)Use a pseudo-random number generator to generate a new random number. Then, verify that this number is not used in a previous block (otherwise generate a new one until no already used number results).(c)Choose the value of the marking property (e.g., entropy) according to the pseudo-random number obtained in the previous step.(d)Build the TSVQ tree with the LL sub-band and verify whether it satisfies the property computed in the previous step. If the value of the property is not verified, report this block as forged. Otherwise, the block is authenticated.


The criterion to be verified is entropy value of each block, which can be checked by using the sequence established in Equation (1). For example, if there are 64 or less blocks in the image, we can use n=6. If the reference value of entropy is R=11, the entropy value to be checked for each block is the following:

Entropy of the block =11−v·h,

where *v* is a 6-bit value between 0 and 63 selected by using the PRNG, and h=1/64 is the quantization step. Thus, a set of entropy values between 11 and 10 is obtained for an image divided into 64 blocks.

Then, using the PRNG sequence g=(5,46,24,62,0,…,30), the following entropy values must be checked for each block:

$$11-\frac{5}{64},11-\frac{46}{64},11-\frac{24}{64},11-\frac{62}{64},11,\ldots ,11-\frac{30}{64}.$$

If the entropy value is verified successfully, the block is then presumed authentic. Contrarily, in case of an unsuccessful verification, the block is identified as manipulated or forged. Thus, the proposed scheme can be used for detecting and locating tampered areas in a watermarked image.

The resolution of the tampering detection varies from 32×32 to 128×128 pixels (in steps of 32 pixels), which corresponds to the block sizes provided by the tiling process.

## 4. Results

As a first step of the scheme, automatic tiling (block division) based on entropy is applied to a “Cuprite” image (as shown in Figure 5). The process groups a few large and small areas, depending on the entropy of each 32 × 32-pixel region. Similar signatures of the hyperspectral image are joined with the neighboring areas. By applying this first step of the scheme, blocks of 64 × 64 pixels of the hyperspectral image with similar entropy are combined in one large block. Finally, a few small-sized blocks of 32 × 32 pixels can be obtained at the bottom and right regions of the image. Similarly, some large-sized blocks of 128 × 128 pixels can be obtained at the center and right parts of the image.

Once the selected blocks are generated, the scheme presented in Section 3 is applied (as shown in Figure 6). The experimental results for this method show an improvement of the previous method [10] with manual block selection. The PSNR values are shown in Table 1 for a “Cuprite” image. The pixel values for this image are represented with 14 bits. For this reason, the equation to compute the PSNR is the following:

$$\mathrm{PSNR}=10\log_{10}\left(\frac{{\left(2^{14}-1\right)}^{2}}{\mathrm{MSE}}\right)=10\log_{10}\left(\frac{{16,383}^{2}}{\mathrm{MSE}}\right),$$

where “MSE” is the mean squared error. For images with 8 bpp, 255 is used instead of 16,383 in the PSNR equation.

Table 1 shows the different values obtained in the watermark embedding process by applying three variants of the DWT (Daubechies 1, 3, and 5). For each method, Table 1 shows the PSNR in decibels, the percentage of Modified Pixels (PMP) and, finally, the mean difference (MD) between the original and the marked values for only modified pixels. The experimental results show a very high value of PSNR (74.55 dB) with only 4.15% PMP (2,436,890 out of total 58,720,256 pixels of the hyperspectral image are modified) and a mean difference of 8.66 units. In this case, the maximum pixel value is reduced to 255 (8 bits per pixel).

In Table 2, the PSNR of each modified band is presented. It can be seen from this table that central bands containing more information exhibit quite high PSNR. The average PSNR value for different modified bands is 61.30 dB. The PMP is very small, i.e., only 32.60% of the 58,720,256 pixels per modified band are modified with a mean difference (Mean diff.) of 17.80 units (where the pixel values range from 0 to 255).

**Figure 8:**
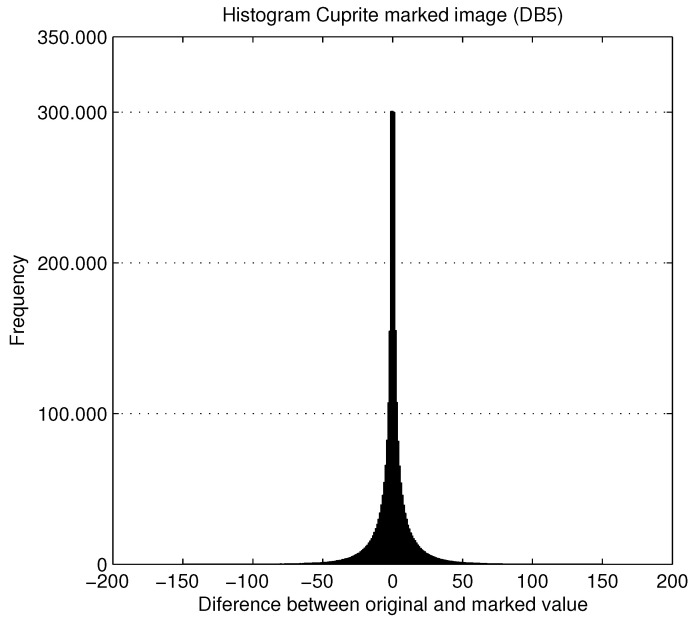
Histogram of the differences between original and marked values for Cuprite image.

Figure 8 shows the histogram of the differences between the original and the marked values for the Cuprite image. It can be seen that fewer values change with a mean difference of 8.66 (where the range of differences of pixel values is from −10 to 10).

**Figure 9:**
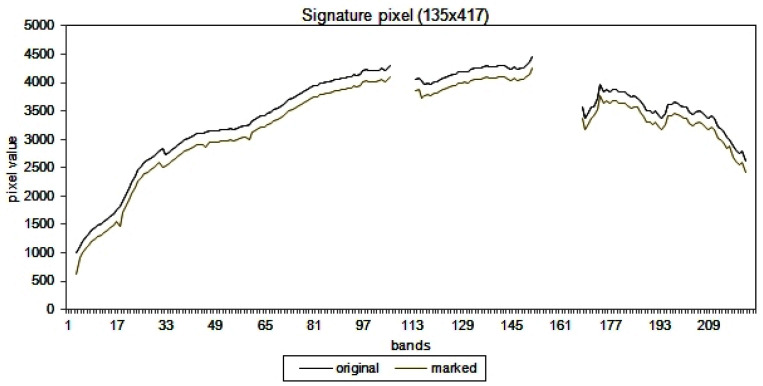
Comparative signatures: Original and marked (shifted down 200 units).

Finally, to show the results of the embedding process, Figure 9 illustrates the difference between the original signature (for all values) and the marked signature of one pixel (135,417). In this figure, all the values for the 224 frequency bands are shifted 200 units down to demonstrate the difference between the two signatures. The figure illustrates that the difference between two signatures is minimal, i.e., the two signatures are almost identical.

### 4.1. Copy-and-Replace Attacks

**Figure 10:**
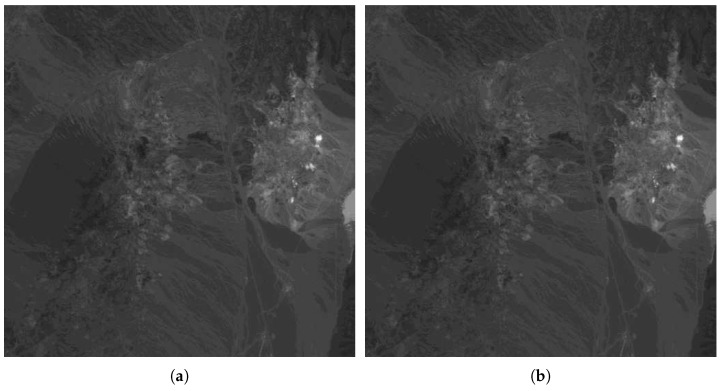
Original (**a**) and marked (**b**) images for band #12.

**Figure 11:**
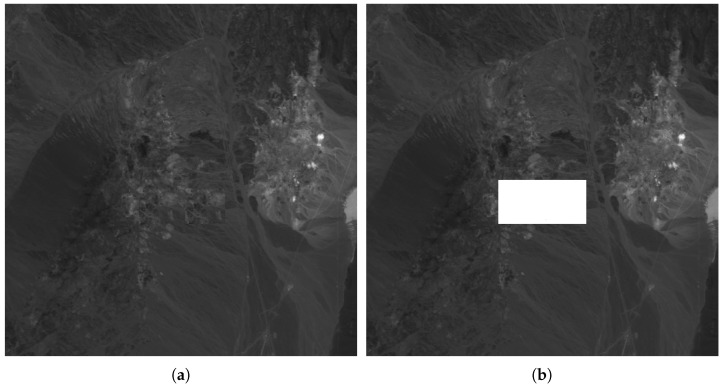
Tampered image (**a**) and tampering detection (**b**) for band #12.

To evaluate the scheme proposed in this article, the robustness against copy-and-replace attack is evaluated. In this attack, part of the image (all bands) is copied to another place. Figure 10 shows the band #12 of the original (Figure 10a) hyperspectral image of a Cuprite area and the marked image (Figure 10b). It can be seen that both images seem to be identical since the difference between the original and the marked band is imperceptible. Figure 11a shows a copy-and-replace attack, in which a rectangular area with all 224 bands is copied to the center of the image. Figure 11b shows the result of the proposed method, and the localization of the tampering area.

Regarding the accuracy of detection and the probability of misdetection, in the experiments we have carried out, there have been no false negatives or false positives. To obtain a false negative (i.e., a forged image that is authenticated as a non-forged one), it is required to replicate the entropy value of each block (which are generated using a secret quantization step and a pseudo-random sequence). For example, the image in Figure 5 is divided into 34 blocks by the block division algorithm. If each block has 64 possible entropy values, the probability that an image satisfies the correct entropy value for all blocks is ${\left(1/64\right)}^{34}\approx 3.9\times 10^{-62}$, which is almost negligible. This illustrates false negatives are almost impossible to occur either by chance or caused by an attacker. In addition, an attacker would not know the block division of the image without having access to the secret key, making it even more difficult to deploy a successful attack that replicates the correct blocks and entropy values for a forged image. Regarding false positives, if an image has been marked with the proposed approach, when no modification occurs, the entropy value of each block must match that selected in the embedding process by means of the secret key and, hence, the watermark will be correctly extracted from the image and no false positives can occur. Only in case of lossy compression (beyond the robustness of the proposed approach) or some kind of signal processing operation, the watermark may not be detected in the image. However, this situation can be considered to be a type of forgery and, hence, should not be considered to be a false positive either. In short, the proposed method can be considered reliable with almost null probabilities of either false positives or false negatives.

### 4.2. Compression Attacks

To evaluate the robustness of the proposed scheme against compression attack, some extra tests are performed using the Kakadu software [36]. All 16 selected bands are compressed and decompressed with JPEG2000 codec. The results, shown in Table 3, illustrate that the embedded mark is resistant to the compression attacks for different values ranging from 8 to 5 bits per pixel (bpp). Compression ratios above 8 bpp are supported, whereas the compression attack eliminates the embedded mark below 5 bpp. In case of higher compression (below 5 bpp), the quality of the compressed image is very low and the resulting image cannot be used due to its poor quality. Such a compression is identified as a tampering attack by the proposed method.

Furthermore, Table 3 presents the mean difference and the maximum difference in the pixel values between the attacked image and the marked one. Also, the last column of Table 3 provides percentage of manipulated samples with respect to the total of each band by this attack.

### 4.3. Comparative Analysis

This section carries out a comparative analysis of the proposed scheme with state-of-the-art watermarking systems for remote sensing images. The comparison, summarized in Table 4, considers the following properties: image type used in the scheme, embedding strategy (single band, multiple bands, or the whole signature), transparency values (in terms of PSNR values), tamper localization (size of detected tampered region), and robustness of the watermarking scheme presented against JPEG2000 compression. In Table 4, a cell contains “No” when the corresponding property is not reported by the watermarking scheme.

From Table 4, the following observations can be made with respect to the selected properties:

- All the schemes are applied to the remote sensing images. In some schemes [11,14], multispectral images are used, while in other systems [10,13,16] including the proposed scheme, hyperspectral images are considered. The scheme proposed by [12] is proposed for raster images, but can be extended to hyperspectral images. Hou et al. [15] proposed a semi-fragile watermarking scheme for panchromatic remote sensing images.
- For the comparison of the PSNR values, the selected watermarking schemes cannot be compared in terms of exactly the same images due to the following reasons: missing exact values of the tuning parameters, usage of different types of images, e.g., raster versus hyperspectral, etc. However, it can be seen, from Table 2 that the proposed method produces similar PSNR values for various images with the same bpp. Therefore, Table 4 shows the comparison of the PSNR values of the selected schemes for the images consisting of 8 bpp.
- It is evident, from Table 4, that the proposed scheme yields extremely high image quality with approximately 65 dB PSNR value for 8 bpp images, higher than the PSNR values obtained by the schemes proposed in [11,12,13] (the best PSNR value among these three schemes is 54 dB). The PSNR value of 65 dB achieved by [14] is similar to the proposed scheme. However, [14] uses homogeneous sites to embed the watermark in a few randomly selected bands. The scheme proposed by [16] achieves 90 dB PSNR value; however, the method is applied to each band individually, which implies that the changes in the signature curves might be uneven, and may result in misclassification of the image. The transparency results of the proposed method are comparable to that of [10].
- The watermarking schemes proposed in [11,12,14,16] do not provide information about tamper localization. The scheme proposed by Serra-Ruiz and Megías [10] successfully identifies intentional tampering and incidental modification of blocks of sizes 64×64 or 32×32. Two schemes proposed in [13,15] can identify smaller tampered areas (8×8 and 4×4, respectively) compared to the proposed scheme, in which the resolution of the tampering detection varies from block sizes of 32×32 to 128×128.
- The method proposed by [10] is not robust against JPEG2000 compression carried out at less than 6 bpp (with an embedding PSNR of 78 dB), whereas the proposed method can survive JPEG2000 compression up to 5 bpp (with embedding PSNR around 75 dB). The scheme proposed in [11] provides robustness against JPEG2000 compression applied to four different images (of size 64 × 64) with an average embedding PSNR around 53.16 dB. The other schemes [12,14,15] provide robustness against JPEG compression instead of JPEG2000 compression, which is much more appropriate than JPEG compression for remote sensing images.

## 5. Conclusions

The Internet provides the facility of distributing multimedia content to many users, who can share images, music or videos with a simple “click” of the mouse on their computer. This makes the techniques of digital content protection of great importance to authenticate it or to protect copyright. This ability to distribute copyrighted works in digital form motivates the need for developing authentication or copyright protection mechanisms.

The article focuses on the development of a method for content authentication and detection of modifications for remote sensing images by selecting the entropy at different regions of these images. The acquisition of remote sensing images is an expensive process, which requires a special sensor (installed on an aircraft or, in some cases, on satellites, such as Landsat or those sent to other planets in NASA or ESA expeditions). This special equipment is used to capture the reflection of light at different wavelengths. Therefore, to preserve the economic values of these images, it is essential to provide content authentication to maintain their integrity. The images obtained from the Earth’s surface or from other planets must be validated to ensure that no part of them has been modified.

In this paper, a semi-fragile watermarking method is presented that embeds a mark in the image content for tampering detection. The proposed method detects the modifications while allowing some lossy compression of the marked image. The results obtained with the proposed method are shown for a specific hyperspectral image, the Cuprite image (a mine from the Nevada state in the USA). The Cuprite hyperspectral image is an AVIRIS type of image with 224 bands with 2 bytes per pixel color depth.

The developed method allows the content provider to mark the image (once) and distribute it with different qualities obtained by applying different compression ratios to it without the removal of the embedded mark. This greatly simplifies the image distribution system and saves the cost of marking different versions of the compressed image with different qualities for different customers. In addition, the image is divided into blocks of varying sizes according to an automatic process determined by the entropy of the pixel blocks. The different sizes make it more difficult for an attacker to try to forge an image.

The method is shown to overcome the properties obtained with other state-of-the-art methods, either in terms of transparency (PSNR) or detection accuracy, or both.

Further research can include using transforms different from the DWT to provide robustness against other attacks, such as JPEG compression instead of JPEG2000. The sizes of the blocks can also be reduced to obtain a more accurate tampering localization.

## Figures and Tables

**Table 1:** PSNR of the marked Cuprite image.

	PSNR (dB)	PMP (%)	MD
DB1	74.52	1.79	15.98
DB3	71.93	3.74	12.42
DB5	74.55	4.15	8.66

**Table 2:** Imperceptibility results of the marked bands (using the db1 integer DWT).

Band #	PSNR	PMP	Mean Diff.	Band #	PSNR	PMP	Mean Diff.
1	60.97	32.72	18.45	9	57.94	32.94	24.80
2	61.30	32.48	16.75	10	62.78	32.50	15.11
3	61.98	32.53	16.03	11	62.70	32.54	15.36
4	62.01	32.61	16.47	12	61.08	32.70	18.79
5	62.14	32.44	16.04	13	61.62	32.68	17.27
6	62.18	32.52	16.06	14	59.86	32.69	19.89
7	62.53	32.50	15.07	15	60.65	32.75	18.96
8	62.08	32.54	15.84	16	58.87	32.87	23.95

**Table 3:** Results of the compression attacks with JPEG2000 for Cuprite image of 14 bpp.

DWT	Compression (bpp)	Resistance of Mark	PSNR (dB)	Mean Difference	Maximum Difference	Manipulated Samples (%)
DB1	8	Yes	85.09	1.19	5	53.80
	7	Yes	80.98	1.56	9	71.85
	6	Yes	76.29	2.38	17	83.81
	5	Yes	70.91	4.13	31	91.26
DB3	8	Yes	85.06	1.19	5	53.90
	7	Yes	80.94	1.56	9	71.94
	6	Yes	76.26	2.39	17	83.89
	5	Yes	70.87	4.15	40	91.28
DB5	8	Yes	85.11	1.19	5	53.58
	7	Yes	81.01	1.55	9	71.72
	6	Yes	76.33	2.38	16	83.74
	5	Yes	70.94	4.13	40	91.23

**Table 4:** Comparative Analysis between the proposed scheme and state-of-the-art watermarking schemes.

Scheme	Image Type	Embedding Strategy	PSNR	Tamper Localization	Robustness against JPEG2000 Compression
Serra-Ruiz and Megías (2011) [10]	Hyperspectral	16 bands of all signatures	~80 dB (14 bpp) ~70 dB (8 bpp)	64×64 (or 32×32)	6 bpp or higher
Fang et al. (2013) [11]	Multispectral	Approximate sub-band	≤ 53 dB (8 bpp)	No	Yes
Chaudhari and Venkatachalam (2014) [12]	Raster (RGB)	2 (LL3 & HH3)	≤ 54 dB (8 bpp)	No	No
Fylakis et al. (2017) [13]	Hyperspectral	RGB	~35 dB (8 bpp)	8×8 blocks	Not reported
Singh (2018) [14]	Multispectral	Selected signatures	~64 dB	No	No
Hou et al. (2018) [15]	Panchromatic	Selected signatures	Not reported	4×4 blocks	No
Carpentieri et al. (2019) [16]	Hyperspectral	Band by band	~90 dB	No	Not reported
Proposed	Hyperspectral	16 bands of all signatures	~75 dB (14 bpp) ~65 dB (8 bpp)	Variable from 32×32 until 128×128	Yes
